# COVID-19 Pandemic in Portugal: Psychosocial and Health-Related Factors Associated with Psychological Discomfort

**DOI:** 10.3390/ijerph19063494

**Published:** 2022-03-15

**Authors:** José Pais-Ribeiro, Alexandra Ferreira-Valente, Margarida Jarego, Elisabet Sánchez-Rodríguez, Jordi Miró

**Affiliations:** 1 Faculty of Psychology and Education Sciences, University of Porto, 4200-135 Porto, Portugal; jlpr@fpce.up.pt; 2 William James Center for Research, ISPA-University Institute, 1100-304 Lisbon, Portugal; mjarego22@gmail.com; 3 Department of Rehabilitation Medicine, University of Washington, Seattle, WA 98104, USA; 4 Universitat Rovira i Virgili, Unit for the Study and Treatment of Pain–ALGOS, Research Center for Behavior Assessment (CRAMC), Department of Psychology, 43007 Tarragona, Spain; elisabet.sanchez@urv.cat (E.S.-R.); jordi.miro@urv.cat (J.M.); 5 Institut d’Investigació Sanitària Pere Virgili, Universitat Rovira i Virgili, 43003 Catalonia, Spain

**Keywords:** COVID-19, psychological discomfort, COVID-19 interference, sociodemographic predictors, psychosocial predictors

## Abstract

The COVID-19 pandemic is a stressful long-lasting event with an increasingly negative impact upon individuals. This study aimed at assessing the magnitude of depression, anxiety, and stress among adults living in Portugal during the first mandatory lockdown of 2020, and the psychosocial and health-related factors associated with these symptoms. A sample of 484 adults (73% women) with an average age of 40 years old (Standard Deviation, SD = 14.03) responded to an online survey. The survey included measures of depression, anxiety, stress, social support, COVID-19 interference in daily life, attitudes towards COVID-19, and health perception. The impact of the lockdown on psychological well-being was large, with up to 36% of the participants showing signs of at least mild psychological discomfort (i.e., depression, anxiety, and stress). Social support, COVID-19 interference on daily life, health perception, and age, explained all the dependent variables. Education level, income, attitudes towards COVID-19, and gender explained some of the dependent variables. These results suggest that the COVID-19 pandemic has a serious impact on the psychological health of Portuguese adults. The role of the procedures to control the pandemic on the mental health of Portuguese adults should not be underestimated.

## 1. Introduction

Exceptional life changes are a source of stress [1,2]. Stressful situations likely cause any type of illness [3]. The COVID-19 pandemic is an exceptional non-normative life change and may be conceptualized as a “cataclysmic event” and a source of “catastrophic secondary stress” [4]. Cataclysmic events are unexpected, uncontrollable, sudden, universal stressors with a severe negative impact on all people simultaneously and with an unpredictable evolution [4]. While the COVID-19 pandemic might not have had a negative impact as severe as the one observed in the case of other cataclysmic events (e.g., war, earthquakes), it shares with such events most of these characteristics. Whereas both primary (i.e., involving stressful events, disasters or emergencies impacting individuals directly, or vicariously, through close ones) and secondary stressors (i.e., stressful long-lasting events with which individuals are not directly—nor vicariously—involved with; these events may be entities in themselves or the consequence of persistent unresolved primary stressors) can be observed in catastrophic situations [5], the pandemic may be best categorized as a secondary stressor: the initial impact was discreet and became worse over time without the individual’s control; thus, a great magnitude of stress, secondary to the COVID-19 pandemic, is expected.

Psychological problems in patients with confirmed or suspected COVID-19, such as depression and anxiety disorders, are highly prevalent in adults and young people worldwide, regardless of cultural background [6,7,8,9,10,11,12,13,14,15,16,17]. These findings have been shown even in the initial stages of the COVID-19 outbreak [11], and psychological discomfort seems to be present in different stages of the COVID-19 pandemic. For instance, a study conducted in China assessed symptoms of depression, anxiety, and stress during the first wave and the second wave [18]. Results revealed moderate-to-severe symptoms of depression (17%), anxiety (29%), and stress (8%), with no significant changes between the first and second wave [18]. Similar findings were found in Italy [19] and the Republic of Ireland [20]. However, it is during the periods of quarantine that individuals reported higher levels of depression, anxiety, and stress [12,13].

Similar findings emerged for the general population living in Portugal during the early stages of this pandemic [14]. The COVID-19 pandemic officially began in Portugal in March 2020 with the identification of the first cases of infected individuals and led to a lockdown that lasted until May of the same year. At that stage, Portugal had a number of cases above the European Union average, though it was much lower than the one observed in the following waves in Portugal [15]. A study conducted between 24 and 27 of March of 2020, indicated that 6% to 12% of the participants (83% women; 31.33 years old, on average; 71% active workers) reported moderate to severe depression, anxiety, and stress, while 49% reported a moderate to severe psychological impact of the COVID-19 outbreak [14], suggesting that further research identifying vulnerable groups that would benefit the most from tailored mental health interventions and policies is necessary.

Sociodemographic, economic, and health-related factors (e.g., gender, age, education, income, pre-COVID-19 mental health) seem to be associated with the severity of psychological discomfort during the COVID-19 pandemic [16,17,21]. Women, younger adults, unemployed individuals, and people with lower education and household incomes reported higher levels of depression, anxiety and stress during mandatory lockdowns associated with COVID-19 [16,17,22,23,24,25,26]. Findings related to the association between sociodemographic and economic variables, on the one hand, and the severity of psychological discomfort, on the other, were not always consistent [27,28]. For example, Lanciano et al. [28] found that even though higher education is a protective factor against perceiving a worse health risk, people with a higher education perceive the socioeconomic and political impact of the COVID-19 pandemic as more severe, which can aggravate their mental health. Further research is, thus, needed to elucidate if previous findings related to the association between sociodemographic, economic, and psychological factors, and levels of psychological discomfort during mandatory lockdowns caused by the COVID-19, generalize across countries, groups, and cultures. This study was aimed at: (a) describing the severity of depression, anxiety, and stress symptoms in the adults living in Portugal during the first COVID-19 lockdown of March through May 2020; and (b) studying the association of sociodemographic, health-related, and psychosocial variables with the severity of depression, anxiety, and stress symptoms in the study’s population.

## 2. Materials and Methods

### 2.1. Participants

The minimum sample size to perform multiple regression was calculated a priori using the G*Power software v. 3.1.9.4 (Universität Kiel, Universität Mannheim, and Universität Düsseldorf, Germany), assuming a medium effect size of 0.15, an alpha level of 0.01, and a power level of 0.95. This resulted in a minimum sample size of 214 participants.

A total of 550 adults agreed to participate in the study, of which 484 provided complete data to be included. Thus, a convenience sample of 484 individuals was used. Inclusion criteria included: (a) being 18 years old or older; (b) living in Portugal during the first mandatory confinement; and (c) agreeing to participate. Most participants were female (73%; *n* = 354), aged between 18 and 73 years old (*M* = 40, *SD* = 14.03). Almost all participants were either single (*n* = 236, 49%), or in a legally recognized conjugal relationship (*n* = 204, 42%). Most individuals had a college degree (Bachelor: *n* = 144, 30%; Master: *n* = 178, 37%; Doctorate: *n* = 56, 12%), and were employed full-time (*n* = 274, 57%). Out of the 309 participants who were (full/part-time) employed, 199 (66%) were teleworking at the time of the first mandatory lockdown in Portugal (see Table 1).

### 2.2. Material

Study participants completed a sociodemographic questionnaire (e.g., gender, age, education level, household income), as well as measures of self-reported health and quality of life, attitude towards COVID-19, social support, COVID-19 interference on daily life activities, and psychological discomfort.

#### 2.2.1. Perceived Health and Quality of Life

Two questions from the 2010–2012 World Values Survey Wave 6 [29] and 2017–2021 World Values Survey Wave 7 [30] were used to assess perceived health and perceived quality of life, respectively, in this study. Participants were asked to rate their perceived health and their perceived quality of life on a Likert scale with scores ranging from 0—“excellent” to 4—“poor”. 

#### 2.2.2. Attitude towards COVID-19

Three independent items assessing the participants’ attitudes towards COVID-19 were developed by the research team for the purposes of this study: (a) perceived severity of COVID-19, ranging from 0—“nothing serious” to 10—“very serious”; (b) perceived risk of being infected with COVID-19, ranging from 0—“none” to 10—“high risk”; and (c) perceived control over COVID-19, ranging from 0—“no control” to 10—“full control”).

#### 2.2.3. Social Support

Participants completed the Intimacy subscale of the Social Support Satisfaction Scale [31]. This subscale is composed of four items with responds in a 5-point Likert scale ranging from “strongly agree” to “totally disagree”. Higher scores indicate greater intimate social support. Previous research supported the reliability of the Intimacy subscale on a sample of Portuguese adults (*α* = 0.74) [31]. This subscale showed acceptable internal consistency in this study (*α* = 0.71).

#### 2.2.4. COVID-19 Interference on Daily Life Activities 

The interference of COVID-19 pandemic on participants’ daily life was assessed through a six-item scale that asked participants to report the magnitude of the pandemic’s interference in six areas of daily life (e.g., “To what extent did the COVID-19 pandemic interfere with your interpersonal relationships with family and friends”) using an 11-point Likert scale ranging from “0” (“Does not Interfere”) to “10” (“Completely Interferes”). The items were developed by the research team for the purpose of this study. The factorial exploration with Promax rotation and scree plot supported a one component factor solution accounting for 54% of the variance on the interference of COVID-19 in daily life. Furthermore, this scale showed good levels of internal consistency (*α* = 0.82) [32].

#### 2.2.5. Psychological Discomfort 

We used the Portuguese version [33] of the 21-items Depression, Anxiety and Stress Scale (DASS-21-P) [34] to assess participants’ level of psychological discomfort. This questionnaire is composed by 21 items grouped in 3 subscales: Depression, Anxiety and Stress. A score per subscale may be computed, higher scores indicating greater depression, anxiety and stress. Differentiated cut-off scores for conventional severity labels—normal, mild, moderate, severe, extremely severe—of depression, anxiety and stress were used [35]. The DASS-21-P has shown to provide valid and reliable scores (0.74 < *α* < 0.85) of depression, anxiety and stress in a sample of Portuguese adults [33]. This measure showed good internal consistency in this study sample (0.87 < *α* < 0.90).

### 2.3. Procedure

This study was approved by the Ethics Committee Ethical Committee for Research from ISPA (reference I/033/04/2020. Data were collected between 1 April 2020, and 2 May 2020, using the Qualtrics online survey platform. Participants were recruited through the dissemination of the study via: (a) website and mailing list of the *Ordem dos Psicólogos Portugueses*; (b) a circular e-mail sent to organizations (e.g., educational and health institutions) and individuals; and (c) social media. The message included a link to an informed consent form and to the online survey. Participants were assured anonymity. Informed consent was obtained for all participants.

#### Data Analysis

First, we computed descriptive statistics with descriptive purposes. Next, to test if depression, anxiety, and stress were associated with gender and age, we used *t*-tests and Pearson correlation coefficients. Finally, to assess the strength of the associations between the severity of psychological discomfort and the sociodemographic, health-related and psychological variables, we performed three stepwise multiple regression analyses with depression, anxiety and stress as dependent variables and sociodemographic variables, health-related variables, attitude towards COVID-19-related variables, social support, and COVID-19 interference on daily life as independent variables. Prior to these analyses, we assessed if the assumptions of these analyses were met: (a) normality of the distributions of the study measures was assessed by computing skewness (*Sk*) and kurtosis (*Ku*), with values of *Sk* and *Ku* lower than 3 and 10, respectively, indicating absence of severe deviance from the normal distribution [36,37]; (b) normality of residuals’ distribution and homoscedasticity of residuals were assessed through normal probability plot of the residuals analysis [38]; (c) Durbin-Watson statistic was computed to assess the independence of errors, with values close to 2 indicating absence of violation of this assumption; (d) variance inflation factor (VIF) for the predictor variables was computed to evaluate multicollinearity, with a VIF lower than 5 suggesting absence of multicollinearity [39]. We used the Statistical Package for Social Sciences for Windows version 27.0 (SPSS Inc., Chicago, IL, USA) to perform all statistical analyses. Alpha was set at 0.05.

## 3. Results

### 3.1. Descriptive Statistic

Most of the study participants perceived their health and quality of life as being at least good (see Table 2). Most participants perceived the severity of and risk of being infected with COVID-19 as high. At least mild levels of depression, anxiety, and stress were reported by 36%, 28%, and 33% of the study participants. Additionally, 9% of the study participants reported severe and extremely severe levels of depression and anxiety, whilst 10% of the participants reported severe or extremely severe levels of anxiety.

### 3.2. Association between Gender, Age, and Psychological Discomfort

No statistically significant differences were found between females and males relative to depression (*t*(480) = −0.097, *p* = 0.923, *d* = −0.01), and anxiety (*t*(480) = −1.63, *p* = 0.104, *d* = −0.168). Female participants reported statistically significant higher stress than male participants (*t*(1480) = −3.47, *p* < 0.001, *d* = −0.414). Age was negatively weakly, but statistically significantly, associated with all three outcome variables (depression: *r* = −0.16, *p* < 0.001; anxiety: *r* = −0.15, *p* < 0.001; and stress: *r* = −0.23, *p* < 0.001). 

### 3.3. Stepwise Multiple Regression Analyses Explaining Psychological Discomfort 

Seven (out of 11) independent variables significantly explained 43% of the variance of depression (see Table 3). These included social support (24%), COVID-19 interference (additional 13%), health perception (additional 2%), age (additional 2%), perceived severity of COVID-19 (additional 0.8%), education level (additional 0.9%), and household’s income (additional 0.7%). 

Six (out of 11) independent variables significantly explained anxiety, explaining 29% of its variance (see Table 4). These include perceived health (13%), COVID-19 interference (6%), social support (4%), education level (2%), household’s income (2%), and age (2%).

Six (out of 11) independent variables significantly contributed to the explanation of stress, accounting for 38% of its variance (see Table 5). These included perceived COVID-19 interference (21%), social support (7%), age (4%), perceived health (3%), household’s income (2%), and gender (1%). 

## 4. Discussion

This study aimed to examine the severity of depression, anxiety, and stress symptoms among adults living in Portugal during the first lockdown and study the association between the former and sociodemographic, health-related, and psychosocial variables. About a third of adults living in Portugal during the first lockdown experienced at least mild levels of psychological discomfort (i.e., depression, anxiety, and/or stress), with women reporting having experienced greater stress than men. Psychological discomfort was most strongly associated with social intimacy, COVID-19 interference on daily life activities, and self-perceived health status. Along with age and household’s income, these three variables significantly explained the severity levels of depression, anxiety, and stress. More importantly, participants reporting higher levels of depression, anxiety, and stress were younger and had higher income, lower levels of social support (intimacy), and worse perceived health. These participants also tended to perceive greater COVID-19 interference with their daily lives. In addition, greater levels of depression and anxiety were also associated with lower education levels, while greater levels of stress were also associated with being female.

Study findings related to the presence of at least mild psychological discomfort (i.e., depression, anxiety, and/or stress) are similar to those of previous studies, using the same questionnaire, and carried out in other countries at the initial phase of the pandemic [40,41,42,43,44,45,46]. In this study, about 30% of the sample showed levels of psychological discomfort above normal. This is especially true for depressive symptoms. These results are not surprising. Psychological issues among the general population are likely when facing a stressful event with worldwide impact and with a high degree of uncertainty related to its development [47,48,49]. Psychological discomfort is also likely to be propelled by imposed restrictions (e.g., limiting social interaction with others). Social interaction and social support are recognized factors that influence both health behaviors and health outcomes [50]. Thus, the impact of social isolation upon psychological health outcomes is not unexpected. The term ‘social distancing’ was applied from the end of February 2020 in a public health context to conquer the coronavirus pandemic [51]. However, a strong discussion was initiated in (social) media, arguing for a change of terminology. However, a rebranding of the term to ‘physical distancing’ was not very successful. In March 2020 the World Health Organization warned of the inappropriate use of the term “social distancing” [52], in a recognition that while maintaining a certain physical separation (i.e., “physical distancing”) from others is key to prevent the spread of the SARS-CoV-2, maintaining a “mental” social proximity is vital to protect mental health [51,52,53]. Our results support the need to distinguish “physical distancing” from “social distancing”, as the level of social intimacy and proximity to close ones was one of the most relevant predictors of psychological discomfort severity.

Our findings suggest the pandemic might have had an important impact on the mental health of adults in the general population. COVID-19 seems to be an important risk factor for mental health disorders which in turn constitute a significant risk factor for the severity of COVID-19 [54]. Thus, it might be necessary to integrate mental health policies and interventions in public health emergencies [26], such as pandemics. Particular attention should be paid to mental health programs as a key strategy to prevent or treat some COVID-19 negative effects. The necessary steps to guarantee the promotion of adequate health care might be creating multidisciplinary teams, safe and clear communication channels, accurate information regarding the pandemic, and mental health counseling online [55]. Governments and public health authorities should provide practical guidelines on how to promote mental health and design interventions for the general public. Moreover, online counseling might be particularly useful during the most critical stages of the pandemic outbreaks. Thus, it might be relevant to promote the development of official, safe, user-friendly, and universally accessible platforms to provide psychological counseling. Moreover, teleworking and studying rooms might benefit people of all ages coming together “virtually”, and strengthening social bonds between individuals. This action could potentially promote social support and decrease the possible negative consequences of teleworking (and “telestudying”). These measures and resources might be especially beneficial for the most vulnerable individuals at greater risk of higher psychological discomfort, which, according to our results and to the findings of previous studies, may be: the young and female, with lower levels of social support, worse perceived health, and a greater perceived COVID-19 interference in daily life activities [7,37,38,51,52,53,54,55].

Some research [56] has shown associations or differences between sociodemographic variables (i.e., age, gender, educational level, income, perception of the severity of COVID-19) and psychological variables, such as depression, anxiety, and stress. These results support our findings, strengthening the notion that sociodemographic variables impact psychological discomfort during the pandemic.

Inconsistent with previous findings [16,17,21], a greater severity of psychological discomfort was observed among adults living in Portugal during the first mandatory lockdown with higher household income. We speculate that this surprising finding may be explained by two non-mutually exclusive reasons. First, it may be argued that individuals with higher incomes are likely to be those in higher-level jobs, which may be already normally subject to daily high levels of stress associated with the demanding organization and management responsibilities of their profession. These levels of psychological discomfort may potentially have been aggravated by the need to accommodate and manage, in a short period, major changes in one’s labor routine (e.g., teleworking), while also (co)managing the implementation of one’s organization/company internal reorganization to implement security measures to prevent the spread of the COVID-19. People from higher-level jobs are most likely than others to have been (co)responsible for the implementation of such contingency measures, while also participating in the management of the negative consequences (e.g., decreased revenue, temporary stop of the production process) of the COVID-19 to companies and organizations. Second, individuals with intermediate- and higher-level jobs are likelier to have transitioned to teleworking, as compared to individuals with lower-level jobs and lower income (e.g., supermarket cashiers). Despite maintaining the daily routine of working in-person might have been associated with stress linked to the daily exposure to the risk of becoming ill, it may be argued that it also represented a smaller disruption to one’s daily routine than transitioning to telework. While maintenance of one’s daily routine may be protective of mental health [57], teleworking may have exposed individuals to greater social isolation and perhaps to increased familiar tension. In addition to a greater smoothness of the boundaries between the time allocated to paid work and personal life—leading, in general, to an extension of working hours—cohabitating teleworking adults were forced to share the same living—and working—space for large periods while their social interactions were mostly limited to other cohabitants. In addition, school shutdowns and in-person classes gave place to remote classes, and children’s and adolescents’ contact with schools and teachers became indirect and mediated by their responsible adult. This delicate work-family balance for individuals in a telework regime, especially those cohabitating with other teleworking adults and with children, may have represented an added burden for these individuals and families, potentially increasing the risk of psychological discomfort among these individuals.

### Limitations

The study limitations include its cross-sectional design, limiting our ability to draw causal conclusions. Second, the study used a non-probabilistic sample, recruited mostly online, and composed mostly of highly educated participants. Thus, the study sample may not be representative of the adult population living in Portugal, limiting the generalizability of the findings. Finally, participants answered the survey online, and online access to the internet may not be equal among groups (e.g., low vs. high socioeconomic status, rural vs. urban areas). Future research should focus on longitudinal studies performed with the same individuals, in order to draw solid conclusions regarding the assessment of psychological discomfort during pandemic states and its associated factors.

## 5. Conclusions

About one-third of the Portuguese adult population experienced at least mild levels of psychological discomfort during the first mandatory lockdown. The psychological impact of the COVID-19 pandemic and of the government measures to tackle it should not be underestimated. Developing psychological interventions targeting the general and vulnerable populations and implementing public mental health strategies from the early stages of this kind of event seems necessary. Particularly vulnerable populations to be targeted by these interventions are those for whom the event seems to interfere with daily lives the most, as well as younger adults with lower education levels, higher income, lower levels of social support, and worse perceived health. Thus, to identify those individuals who might need and benefit the most from such interventions, a longitudinal assessment of individuals’ psychological discomfort, social support, and COVID-19 interference with their daily life over time is warranted. 

## Figures and Tables

**Table 1:** Study participants characterization.

	*N*	*%*	*M*	*SD*	*Sk*	*Ku*
Sex (Female)	354	73	-	-	-	-
Age	-	-	39.98	14.03	0.270	−0.974
Marital Status			-	-	-	-
Single	236	49				
Legally recognized conjugal relationship	204	42				
Separated/Divorced	38	8				
Widow	6	1				
Education Level (ISCED 2011) *			-	-	-	-
Level 1 or lower	8	2				
Level 2	9	2				
Level 3	89	18				
Levels 6 and 7	322	66				
Level 8	56	12				
Working Status			-	-	-	-
Full-time worker	274	57				
Part-time worker	35	7				
Unemployed	32	7				
Retired	29	6				
Student	89	18				
Other	25	5				
Teleworking (Yes)	199	66	-	-	-	-
Household Income (in euros)	-	-	2696.26	2234.51	2.71	10.37

*Note*: *n*—Number of participants; *%*—Percentage; *M*—Mean; *SD*—Standard deviation; *Sk*—Skewness; *Ku*—Kurtosis; * ISCED 2011—2011 International Standard Classification of Education.

**Table 2:** Descriptive statistics.

	*n*	*%*	*M*	*SD*	*Sk*	*Ku*
Perceived Health (0–4)	-	-	1.62	1.00	−0.077	−0.665
Perceived Quality of life (0–4)	-	-	2.13	0.963	−0.463	−0.240
Perceived severity of COVID-19 (0–10)	-	-	8.12	1.76	−1.14	1.40
Perceived risk of being infected with COVID-19 (0–10)	-	-	6.45	2.27	−0.260	−0.541
Perceived control over COVID-19 (0–10)	-	-	5.25	2.28	−0.538	−0.256
Social Support-Intimacy Subscale	-	-	14.56	3.93	−0.443	−0.481
DASS-21 Depression	-	-	8.62	8.55	1.34	1.72
Normal	311	64	-	-	-	-
Mild	53	11	-	-	-	-
Moderate	75	16	-	-	-	-
Severe	24	5	-	-	-	-
Extremely severe	21	4	-	-	-	-
DASS-21 Anxiety	-	-	5.56	7.31	2.09	4.94
Normal	349	72	-	-	-	-
Mild	33	7	-	-	-	-
Moderate	57	12	-	-	-	-
Severe	14	3	-	-	-	-
Extremely severe	31	6	-	-	-	-
DASS-21 Stress	-	-	12.69	8.55	0.80	0.62
Normal	327	68	-	-	-	-
Mild	58	12	-	-	-	-
Moderate	54	11	-	-	-	-
Severe	33	7	-	-	-	-
Extremely severe	12	3	-	-	-	-

*Note: n*—Number of participants; *%*—Percentage; *Me*—Median; *M*—Mean; *SD*—Standard deviation; *Sk*—Skewness; *Ku*—Kurtosis.

**Table 3:** Stepwise multiple regression analysis explaining depression.

	*R*	*R* ^2^ *_a_*	Δ*R*^2^	*B*	*β*	*t*
Step 1	0.5	0.24	0.24			
Intimacy				−1.035	−0.49	−11.66 ***
Step 2	0.61	0.37	0.13			
Intimacy						
COVID-19 Interference				2.99	0.36	9.08 ***
Step 3	0.63	0.39	0.02			
Intimacy						
Perceived COVID-19 Interference						
Perceived Health				1.309	0.33	3.91 ***
Step 4	0.64	0.41	0.02			
Intimacy						
Perceived COVID-19 Interference						
Perceived Health						
Age				−0.09	−0.02	−4.08 ***
Step 5	0.65	0.42	0.008			
Intimacy						
Perceived COVID-19 Interference						
Perceived Health						
Age						
Perceived Severity of COVID-19				−0.45	−0.09	−2.44 *
Step 6	0.66	0.42	0.009			
Intimacy						
Perceived COVID-19 Interference						
Perceived Health						
Age						
Perceived Severity of COVID-19						
Education Level				−0.71	−0.10	−2.57 *
Step 7	0.66	0.43	0.007			
Intimacy						
Perceived COVID-19 Interference						
Perceived Health						
Age						
Education Level						
Perceived Severity of COVID-19						
Household’s Income				<0.001	0.09	2.28 *

*Note*: *R*—Multiple correlation coefficient; *R*^2^_a_*—* Adjusted squared multiple correlation; Δ*R*^2^*—*Change in *R*^2^; *B*—Coefficients; *β—*Standardized regression coefficients; *t—t*-test value. * p < 0.05; *** *p* < 0.001.

**Table 4:** Stepwise multiple regression analysis explaining anxiety.

	*R*	*R* ^2^ *_a_*	Δ*R*^2^	*B*	*β*	*t*
Step 1	0.36	0.13	0.13			
Health Perception				2.54	0.36	7.87 ***
Step 2	0.44	0.19	0.06			
Perceived Health						
Perceived COVID-19 Interference				1.86	0.26	5.43 ***
Step 3	0.48	0.23	0.04			
Perceived Health						
Perceived COVID-19 Interference						
Intimacy				−0.35	−0.20	−4.43 ***
Step 4	0.5	0.25	0.02			
Perceived Health						
Perceived COVID-19 Interference						
Intimacy						
Education Level				−0.93	−0.15	−3.54 ***
Step 5	0.52	0.27	0.02			
Perceived Health						
Perceived COVID-19 Interference						
Intimacy						
Education Level						
Household’s income				<0.001	0.15	−3.46 ***
Step 6	0.54	0.29	0.02			
Perceived Health						
Perceived COVID-19 Interference						
Intimacy						
Education Level						
Household’s income						
Age				−0.07	−0.15	3.46 ***

*Note*: *R*—Multiple correlation coefficient; *R*^2^_a_*—*Adjusted squared multiple correlation; Δ*R*^2^*—*Change in *R*^2^; *B*—Coefficients; *β—*Standardized regression coefficients; *t—t*-test value. *** *p* < 0.001.

**Table 5:** Stepwise multiple regression analysis explaining stress.

Predictors	*R*	*R* ^2^ *_a_*	Δ*R*^2^	*B*	*β*	*T*
Step1	0.47	0.21	0.21			
Perceived COVID-19 Interference				3.9	0.47	10.78 ***
Step 2	0.53	0.28	0.07			
Perceived COVID-19 Interference						
Intimacy				−0.55	−0.26	−6.25 ***
Step 3	0.57	0.32	0.04			
Perceived COVID-19 Interference						
Intimacy						
Age				−0.12	−0.20	−4.89 ***
Step 4	0.59	0.35	0.03			
COVID-19 Interference						
Intimacy						
Age						
Perceived Health				1.58	0.19	5.53 ***
Step 5	0.61	0.37	0.02			
Perceived COVID-19 Interference						
Intimacy						
Age						
Perceived Health						
Household’s income				0.001	0.15	3.86 ***
Step 6	0.62	0.38	0.01			
Perceived COVID-19 Interference						
Intimacy						
Age						
Perceived Health						
Household’s income						
Gender				−2.06	−0.11	2.82 **

*Note*: *R*—Multiple correlation coefficient; *R*^2^_a_*—* Adjusted squared multiple correlation; Δ*R*^2^*—*Change in *R^2^*; *B*—Coefficients; *β—*Standardized regression coefficients; *t—t*-test value. ** p < 0.01; *** *p* < 0.001.

## Data Availability

The data presented in this study is available on request from the corresponding author.
